# Integrative Multi-Omics Analysis Characterizes Immune Dysregulation and Altered CD4^+^ Central Memory T-Cell Abundance in Allergic Rhinitis

**DOI:** 10.3390/biomedicines14071541

**Published:** 2026-07-09

**Authors:** Aodeng Surita, Tianhui Kang, Chuan Chen, Hong Qiao, Wei Lv, Yang Zha

**Affiliations:** Department of Otolaryngology-Head and Neck Surgery, Peking Union Medical College Hospital, Chinese Academy of Medical Sciences and Peking Union Medical College, Beijing 100730, China; aodengsurita@pumch.cn (A.S.); kangth0721@126.com (T.K.); chenchuan_doct@163.com (C.C.); qiaoh19@student.pumc.edu.cn (H.Q.)

**Keywords:** allergic rhinitis, immunomodulatory score, CD4^+^ central memory T cells, immune dysregulation, multi-omics, drug repositioning

## Abstract

**Background/Objectives**: Allergic rhinitis (AR) is a highly prevalent chronic inflammatory disease of the upper airway characterized by immune dysregulation. This study aimed to systematically characterize the immunomodulatory landscape of AR and identify exploratory molecular and cellular features associated with disease-related immune remodeling. **Methods**: Public bulk transcriptomic datasets and single-cell RNA sequencing data were integrated to identify immunomodulatory-related differentially expressed genes (IMRDEGs), construct an immunomodulatory score (IM.Score), evaluate immune cell infiltration, and characterize cell-type composition and intercellular communication. Machine learning was used to derive an exploratory molecular signature, and L1000CDS2-based drug repositioning analysis was performed to identify in silico candidate compounds predicted to oppose AR-associated transcriptional signatures. **Results**: A total of 12 IMRDEGs were identified and used to construct the IM.Score, which was lower in AR samples relative to control samples. Immune infiltration analysis demonstrated that IM.Score stratification was associated with distinct immune microenvironmental profiles. Single-cell analysis revealed potential reductions in CD4^+^ central memory T cells, which represent an immune cellular alteration requiring further experimental verification, alongside attenuated intercellular communication involving this cell population. A six-gene exploratory molecular signature comprising *NFKBIA*, *PDCD1*, *MYC*, *IFNG*, *FOXP3*, and *CD274* showed favorable performance in the training cohort (AUC = 0.992, 95% CI: 0.974–1.000) but failed to generalize in the external validation cohort (AUC = 0.500, 95% CI: 0.245–0.755), precluding clinical diagnostic interpretation at this stage. Drug repositioning analysis identified candidate compounds, including narciclasine and BRD-K91370081, with the potential to reverse AR-associated transcriptional alterations. **Conclusions**: This integrative multi-omics analysis identifies coordinated molecular, cellular, and communication-level immune alterations in AR. The IM.Score, altered CD4^+^ central memory T-cell abundance, and six-gene exploratory molecular signature may be regarded as hypothesis-generating candidate clues pending further experimental and clinical validation.

## 1. Introduction

Allergic rhinitis (AR) is a highly prevalent chronic inflammatory disorder of the upper airway, affecting approximately 10–40% of the global population and imposing a substantial socioeconomic burden [1,2]. Clinically, AR is characterized by nasal itching, rhinorrhea, nasal congestion, and sneezing, which significantly impair quality of life, sleep, and work productivity. Although current therapeutic strategies, including antihistamines and intranasal corticosteroids, could alleviate symptoms in most patients, a considerable proportion of individuals experience suboptimal responses, frequent recurrence, or disease persistence [3]. These clinical challenges highlight the need to clarify AR pathogenesis and to develop better molecular stratification and therapeutic exploration strategies.

AR is widely recognized as an immune-mediated disease involving complex interactions among epithelial barrier dysfunction, allergen-specific IgE responses, type 2 inflammatory pathways, cytokine signaling, and immune regulatory networks [4,5]. Previous studies have identified multiple immune-related mediators implicated in AR pathogenesis, including cytokines, transcriptional regulators, immune checkpoint molecules, and T-cell-associated regulatory factors such as *IFNG*, *TGFB1*, *FOXP3*, and *PDCD1* [6,7,8]. These findings have advanced the understanding of allergic inflammation and immune tolerance in AR. However, most available studies have focused on selected immune molecules, specific pathways, or single-layer transcriptomic profiles. Therefore, an integrated analysis linking immunomodulatory gene expression, immune cell composition, and intercellular communication may provide a more comprehensive view of immune dysregulation in AR [9].

This study aimed to identify immunomodulatory-related differentially expressed genes, construct a quantitative immunomodulatory score, characterize immune cell remodeling, and explore candidate molecular signatures and in silico drug-repositioning clues. This integrative framework may help link gene-level dysregulation with immune cell heterogeneity and intercellular communication changes, thereby providing hypothesis-generating insights into immune dysregulation and precision-oriented research in AR.

## 2. Methods

**Figure 1:**
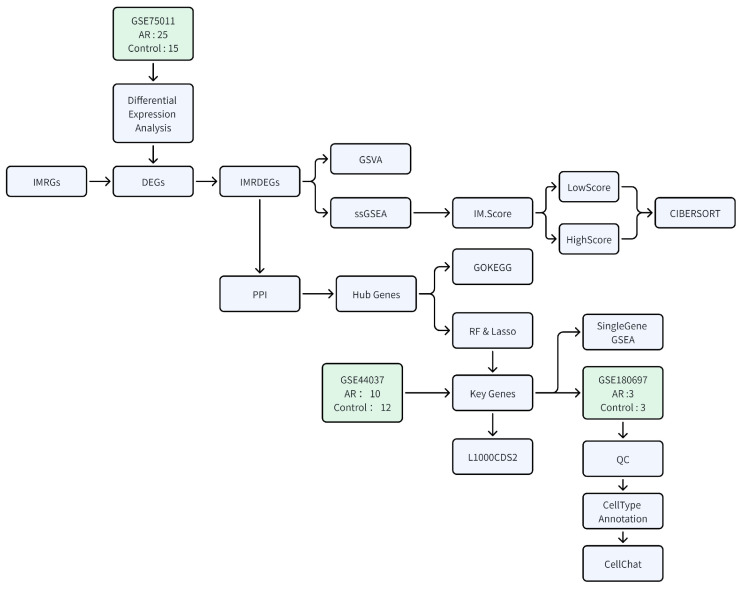
Overall analytical workflow of this study. AR, allergic rhinitis; IM, immunomodulatory; IMRGs, immunomodulatory-related genes; DEGs, differentially expressed genes; IMRDEGs, immunomodulatory-related differentially expressed genes; GSVA, gene set variation analysis; ssGSEA, single-sample gene set enrichment analysis; CIBRSORT, cell-type identification by estimating relative subsets of RNA transcripts; PPI, protein–protein interaction; GO, Gene Ontology; KEGG, Kyoto Encyclopedia of Genes and Genomes; RF, Random Forest; Lasso, least absolute shrinkage; GSEA, gene set enrichment analysis and selection operator; QC, quality control.

The overall analytical workflow is illustrated in Figure 1.

### 2.1. Data Acquisition

Three public transcriptomic datasets associated with AR were retrieved from the Gene Expression Omnibus (GEO) database. Bulk dataset GSE75011 (25 AR patients, 15 controls; peripheral blood) was profiled on platform GPL16791, and bulk dataset GSE44037 (10 AR patients, 12 controls; nasal epithelial cells) was used on platform GPL13158. These two bulk cohorts were used for transcriptome analysis, while the single-cell RNA-seq dataset GSE180697 (3 AR patients, 3 controls; peripheral blood mononuclear cells) was applied for cellular profiling and analyzed separately without batch combination with bulk data. Probe-to-gene mapping was completed relying on official annotation files of the corresponding GEO platforms. Probes that could not be matched to official gene symbols were directly discarded. For multiple probes targeting one identical gene symbol, we adopted the average expression value to represent the final expression level of this gene. All raw expression matrices underwent log2 scale inspection before subsequent processing. Raw data without log2 transformation were converted with log2(x + 1), whereas matrices already on log2 scale were kept unchanged to avoid repeated transformation. The bulk expression matrices of GSE75011 and GSE44037 were normalized separately. The normalizeBetweenArrays function in the R package limma (version 3.62.1) was used to reduce technical biases across samples. Boxplots and principal component analysis (PCA) were generated to evaluate the normalization performance. Boxplots illustrated global gene expression distribution shifts between the two datasets before and after correction. PCA plots colored by dataset revealed prominent batch separation in unprocessed raw data, which was largely eliminated after between-array normalization, confirming effective removal of dataset-specific technical variation. All 40 samples from GSE75011 and all 22 samples from GSE44037 were retained for downstream analysis after quality control; no samples were excluded due to poor quality metrics.

**Figure 2:**
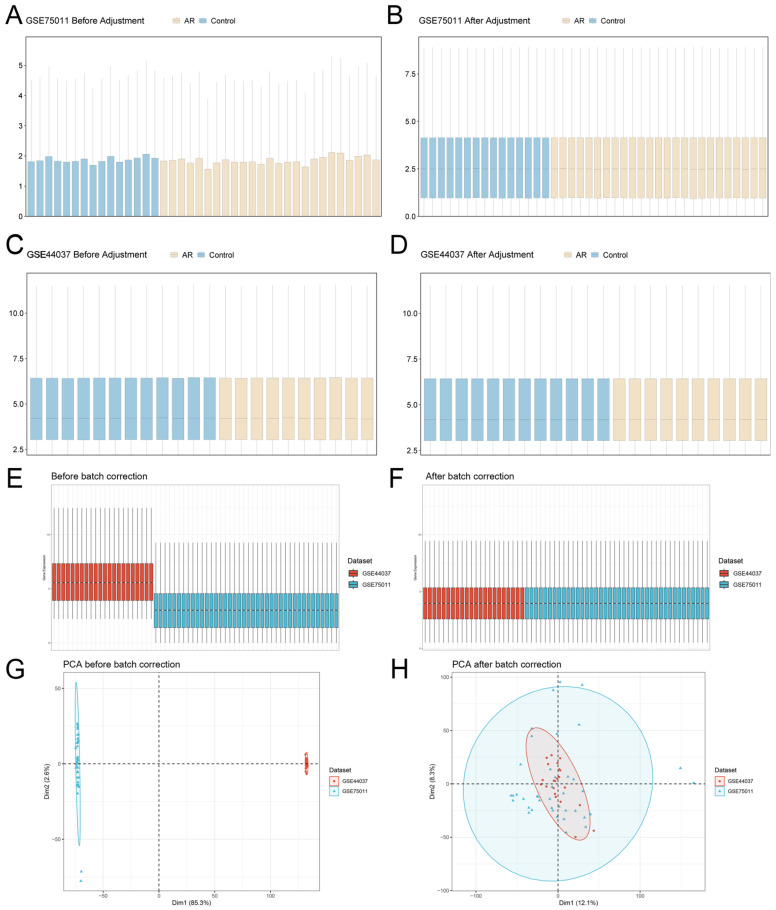
Batch normalization evaluation of two bulk transcriptomic datasets used for model training and external validation. Gene expression distribution boxplots before and after between-array normalization in the training cohort GSE75011 (peripheral blood, AR *n* = 25, control *n* = 15) (**A**,**B**) and external validation cohort GSE44037 (nasal epithelial cells, AR *n* = 10, control *n* = 12) (**C**,**D**). (**E**,**F**) Combined gene expression distribution boxplots of GSE75011 and GSE44037 before and after cross-dataset batch correction, colored by dataset (red: GSE44037, cyan: GSE75011). (**G**,**H**) Principal component analysis (PCA) scatter plots showing sample clustering patterns before and after cross-dataset batch correction. (**G**) Severe separation between the two datasets, driven by technical batch effects, was observed in raw data; (**H**) partial sample integration was achieved after batch adjustment. AR, allergic rhinitis, and PCA, principal component analysis.

After batch correction, the gene expression distributions were well-balanced across groups, and PCA results confirmed substantial mitigation of inter-study batch effects (Figure 2).

### 2.2. Identification of IMRDEGs

Immunomodulatory-related genes (IMRGs) were collected from GeneCards (relevance score > 3, protein-coding) and MSigDB. Differential expression analysis was performed using the limma package. BenjaminiHochberg-adjusted *p*-values were calculated. Differentially expressed genes (DEGs) in GSE75011 were identified using limma with thresholds |logFC| > 0.262 and *p* < 0.05. The threshold |log2FC| > 0.262 corresponds to a fold change greater than 1.2. Given that this study focuses on subtle transcriptional shifts linked to immune regulation, immune-associated genes often exhibit moderate expression alterations rather than drastic fold changes. This mild threshold was adopted to retain candidate immune-relevant transcripts, and subsequent intersection with curated immunomodulatory gene sets further refined the output to enrich biologically relevant signals. Immunomodulatory-related differentially expressed genes (IMRDEGs) were obtained by intersecting DEGs with IMRGs and visualized with volcano plots and heatmaps.

### 2.3. Functional Enrichment Analysis

Gene set variation analysis (GSVA) was performed using the c2.cp.v2023.2.Hs.symbols gene set to compare pathway enrichment between AR and control samples. Gene Ontology (GO) and Kyoto Encyclopedia of Genes and Genomes (KEGG) enrichment analyses of hub genes were conducted using clusterProfiler, with significant terms defined as p.adjust < 0.05 and false discovery rate (FDR) < 0.25.

### 2.4. Construction of IM.Score

To quantify the immunomodulatory state at the individual sample level, an immunomodulatory score (IM.Score) was calculated using single-sample gene set enrichment analysis (ssGSEA) based on IMRDEGs. The normalized expression matrix of GSE75011 was used as input, and the 12 immunomodulatory-related differentially expressed genes were assembled into a predefined gene set. The ssGSEA algorithm embedded in the GSVA R package was adopted to compute the enrichment score of each sample, which was defined as the IM.Score. The mathematical formula for IM.Score calculation is presented as IM.Score*_i_* = ssGSEA*_i_* (G_IMRDEG_), where *i* represents the *i*-th sample, and G_IMRDEG_ denotes the gene set composed of the 12 IMRDEGs. No extra weight was assigned to individual genes during calculation; upregulated and downregulated genes were not separated into independent directional gene sets, and no reverse weighting was applied to transcripts. All enrolled genes participated in scoring uniformly as an integrated gene set. Subsequently, AR samples were stratified into the HighScore subgroup and the LowScore subgroup according to the median IM.Score value for subsequent immune infiltration evaluation and differential expression analysis of candidate genes. This approach allows transformation of gene-level variation into a sample-level immune regulatory metric.

### 2.5. Immune Infiltration Analysis

The proportion of 22 immune cell types was estimated using the CIBERSORT algorithm. A total of 25 samples were retained for the final CIBERSORT analysis. The *p*-value, correlation coefficient, and root mean square error (RMSE) generated by CIBERSORT were adopted to evaluate the deconvolution confidence of each individual sample. CIBERSORT was run with the LM22 signature matrix and 1000 permutations; only samples with CIBERSORT *p* < 0.05 were retained. Differences in immune infiltration between high- and low-IM.Score subgroups were analyzed, and correlations between IMRDEGs and immune cells were determined by the Spearman correlation.

### 2.6. Machine Learning-Based Exploratory Molecular Signature

A protein–protein interaction (PPI) network of IMRDEGs was constructed via STRING with a minimum interaction score > 0.7. All 12 IMRDEGs were directly used as input for machine learning. Random Forest (RF) and least absolute shrinkage and selection operator (LASSO) regression with 10-fold cross-validation were applied to establish a diagnostic model in the training cohort (GSE75011, peripheral blood samples). The model was further validated in an independent external cohort (GSE44037, nasal epithelial cells) using the coefficient matrix derived from the training set. Model performance was evaluated by receiver operating characteristic (ROC) curves, nomogram, and decision curve analysis (DCA). Area under the curve (AUC) values with 95% confidence intervals (95% CI) were calculated to assess discriminatory ability in both cohorts.

### 2.7. Single-Cell RNA-Seq Analysis

Single-cell data was processed using the Seurat package for quality control, normalization, PCA, and t-distributed stochastic neighbor embedding (t-SNE) clustering. The single-cell dataset GSE180697 contained 3 AR patients and 3 healthy control donors. The mitochondrial read proportion threshold was set to <25%, and the clustering resolution was fixed at 0.6. After cell type annotation, the cell count and proportion of each cell subset were calculated per individual donor to evaluate inter-individual variation. Genes expressed in fewer than 10 cells were filtered out to exclude low-quality features. Cell types were annotated using SingleR with the Human Primary Cell Atlas database. Per-donor post-QC cell numbers, median genes per cell, median UMI counts, median mitochondrial read fractions, and the count and percentage of CD4^+^ central memory T cells for each sample were summarized in Supplementary Table S1.

### 2.8. Cell–Cell Communication Analysis

Cell–cell communication networks were inferred using the CellChat package (version 1.6.1) based on CellChatDB human ligand–receptor database CellChatDB.human. Separate CellChat models were constructed for the AR group and the control group, respectively. The computeCommunProb function was applied to calculate communication probabilities of each ligand–receptor pair with raw.use = TRUE. *p*-values were calculated via the default permutation test embedded in CellChat with 100 permutations. The filterCommunication function was used to screen valid communication links, where min.cells was set to 10 to filter out cell clusters with insufficient cell counts. Only communication pairs with *p* < 0.05 were retained as significant interactions. No cell population downsampling was performed for either group, and no extra normalization steps for cell abundance were applied during the whole analysis workflow. Communication patterns were compared between the AR and control groups, with a focus on CD4^+^ central memory T cells.

### 2.9. Drug Repositioning

Drug repositioning was performed using the L1000CDS2 platform with DEGs as input signatures [10]. Differentially upregulated and downregulated genes derived from the AR versus control comparison in the GSE75011 dataset were separately used as disease-associated transcriptional input signatures for compound screening. The top 50 ranked perturbation records returned by L1000CDS2 were sorted by connectivity score, and core output fields including drug name, score, overlap, cell line, dose, time and signature ID were extracted for subsequent filtering. The interaction ratio was defined as the proportion of key immune regulatory genes with predicted reversed expression changes induced by the compound relative to the total number of key genes. Only compounds with an interaction ratio greater than 30% were retained as candidates in this study. All qualified compounds were ranked by connectivity score based on their capacity to reverse AR-associated transcriptional profiles, and those showing the strongest reversal potential were selected for subsequent analysis.

### 2.10. Statistical Analysis

All statistical analyses were performed using R (version 4.3.1). Continuous variables were compared using the Wilcoxon rank-sum test unless otherwise specified. Correlation analyses were conducted using Spearman’s rank correlation. Multiple-testing correction was implemented for all enrichment analyses to calculate adjusted *p*-values and FDR. *p* < 0.05 was considered statistically significant.

## 3. Results

### 3.1. Identification of Immunomodulatory Gene Signatures Underlying Allergic Rhinitis

Differential gene expression analysis was performed using the GSE75011 dataset. A total of 1238 DEGs were identified, including 534 upregulated and 704 downregulated genes.

By intersecting DEGs with curated IMRGs, 12 IMRDEGs were identified, including *TNF*, *IFNG*, *CASP3*, *TGFB1*, *CD274*, *FOXP3*, *PDCD1*, *MYC*, *NFKBIA*, *IL2RB*, *TNFSF10*, and *NLRP3*. Expression pattern analysis showed distinct dysregulation between AR and control samples.

**Figure 3:**
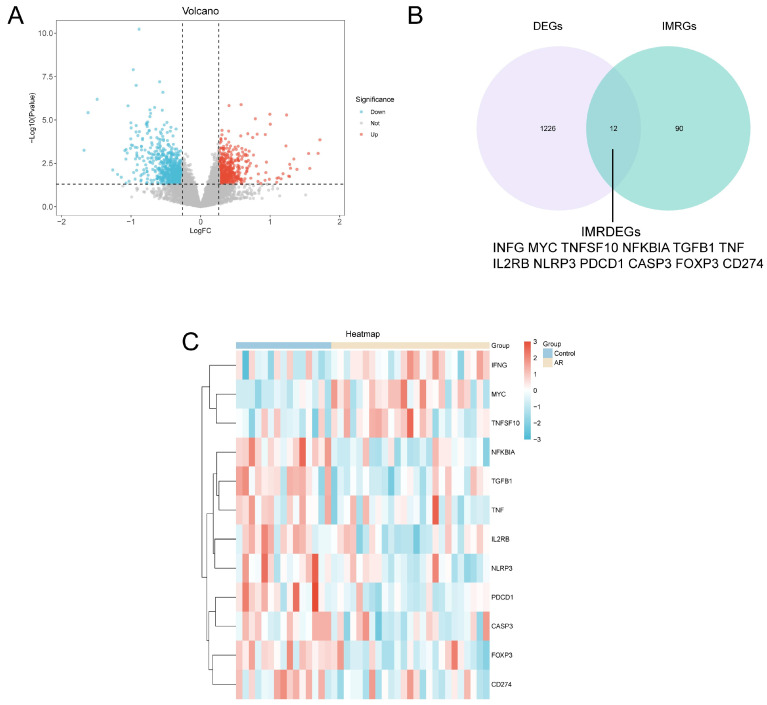
Identification of 12 immunomodulatory-related differentially expressed genes in AR (GSE75011; AR *n* = 25, control *n* = 15). (**A**) Volcano plot of differentially expressed gene analysis between AR and control. The vertical and horizontal dashed lines correspond to the cut-off thresholds of |logFC| = 0.262 and *p* = 0.05, respectively. (**B**) Venn diagram of DEGs and immunomodulation-related genes (IMRGs). (**C**) Heat map of IMRDEGs. AR, allergic rhinitis; DEGs, differentially expressed genes; IMRGs, immunomodulatory-related genes; IMRDEGs, immunomodulatory-related differentially expressed genes.

In total, 12 IMRDEGs were identified and visualized by volcano plot and heatmap (Figure 3).

### 3.2. IM.Score Characterizes Immunomodulatory Transcriptional Difference in Allergic Rhinitis

The IM.Score was constructed based on the expression profiles of IMRDEGs using the ssGSEA algorithm. The IM.Score derived from the 12 IMRDEGs was applied to quantify the immunomodulation-associated transcriptional signature of each sample. AR samples were further divided into the high-score group and the low-score group based on the median IM.Score value across all AR patients, which was used to compare candidate gene expression patterns and immune-cell compositional differences. Comparative analysis demonstrated that the IM.Score was lower in AR samples compared with healthy controls (*p* < 0.05).

ROC curve analysis showed that IM.Score distinguished AR from control samples with moderate-to-high diagnostic accuracy. Stratification by IM.Score divided AR samples into high-score and low-score subgroups with different expression patterns of the 12 IMRDEGs.

**Figure 4:**
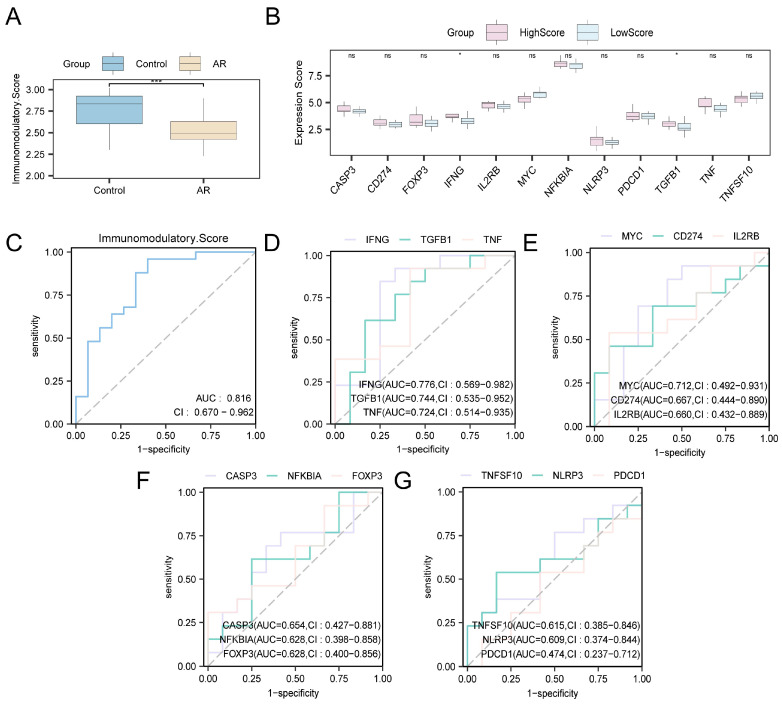
Construction and diagnostic performance of the IM.Score. (**A**) IM.Score in AR and control groups. (**B**) Expression of 12 IMRDEGs in high- and low-IM.Score subgroups. (**C**–**G**) ROC curves with AUC values for IM.Score and individual IMRDEGs. The dashed lines represent the reference line of AUC = 0.5 (random diagnostic performance). IM.Score, immunomodulatory score; AR, allergic rhinitis; ROC, receiver operating characteristic; AUC, area under the curve; CI, confidence intervals; ns, *p* ≥ 0.05; * *p* < 0.05; *** *p* < 0.001.

Construction and exploratory AR–control separation of the IM.Score are shown in Figure 4.

### 3.3. Immune Infiltration Landscape Is Associated with IM.Score-Defined Subgroups in Allergic Rhinitis

Immune cell infiltration analysis was conducted using the CIBERSORT algorithm to estimate the proportions of 22 immune cell subsets in AR samples. Substantial heterogeneity in immune cell composition was observed across samples. The final CIBERSORT analysis included 25 samples. All samples exhibited a CIBERSORT *p*-value equal to 0, satisfying the confidence threshold of *p* < 0.05. The median sample correlation coefficient was 0.794 (range: 0.549–0.857), and the median RMSE was 0.610 (range: 0.522–0.849).

**Figure 5:**
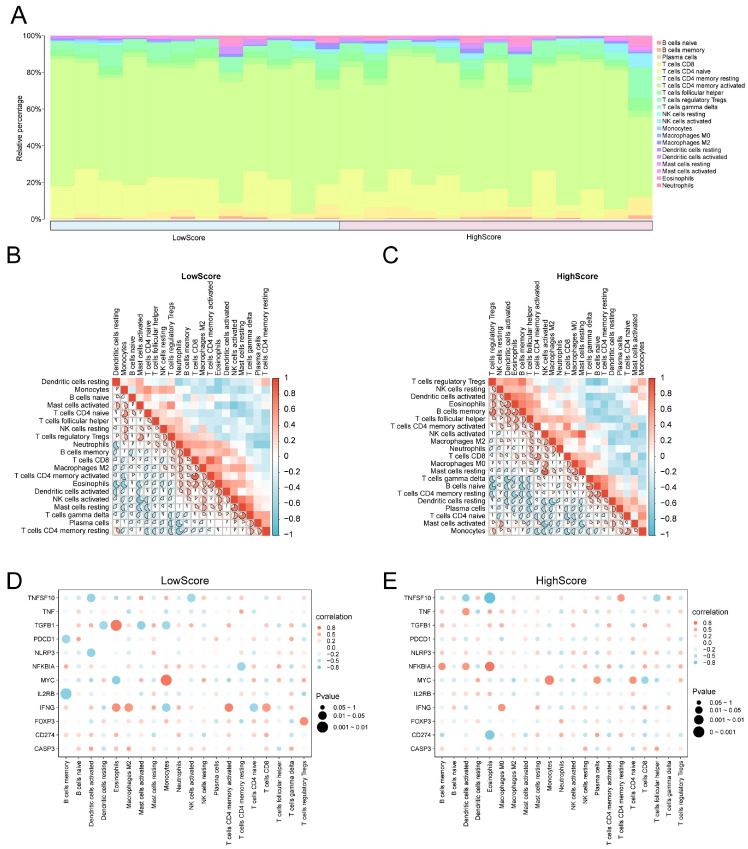
Immune infiltration landscape in AR stratified by IM.Score. (**A**) Relative proportions of 22 immune cell subsets. All included samples met the CIBERSORT confidence criterion of *p* < 0.05. (**B**,**C**) Correlation heatmaps illustrating the pairwise correlations among infiltrating immune cells in the low-IM.Score (**B**) and high-IM.Score (**C**) subgroups, with color intensity indicating the strength of correlation (red: positive correlation, and blue: negative correlation). (**D**,**E**) Bubble plots depicting the correlations between the 12 immune-related differentially expressed genes (IMRDEGs) and infiltrating immune cells in the low-IM.Score (**D**) and high-IM.Score (**E**) subgroups. The color of each bubble represents the correlation coefficient, and the size represents the significance level (*p*-value). AR, allergic rhinitis; IM.Score, immunomodulatory score.

AR samples were divided into high-IM.Score and low-IM.Score subgroups. Differences in the abundance of multiple immune cell populations were observed between the two subgroups. Correlation analysis revealed distinct correlation patterns among immune cell subsets in each subgroup. Additionally, significant correlations were detected between the 12 IMRDEGs and specific immune cell populations (Figure 5).

### 3.4. Single-Cell Analysis Suggests Altered CD4^+^ Central Memory T-Cell Abundance in Allergic Rhinitis

**Figure 6:**
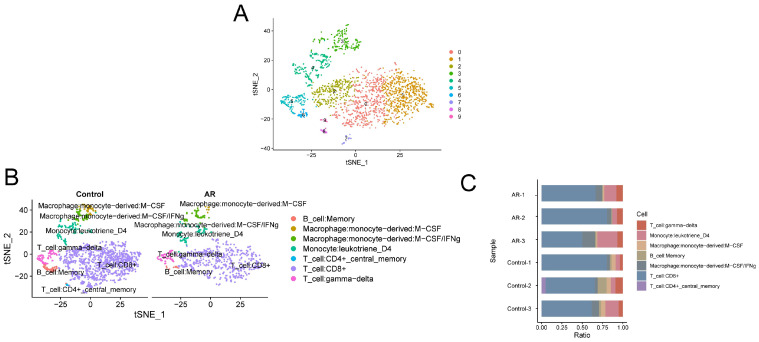
Single-cell RNA-seq analysis of peripheral immune cells from AR patients and healthy controls (GSE180697; AR *n* = 3, control *n* = 3). (**A**) t-SNE visualization of all single cells colored by the identified cell clusters (0–9). A t-SNE dimensionality reduction plot showing the clustering of all PBMC from the GSE180697 dataset. (**B**) t-SNE visualization of annotated immune cell types in healthy control and AR samples. t-SNE plots showing the distribution of major immune cell populations in the control group and AR group. (**C**) Stacked bar plot showing the proportions of annotated cell types across individual samples. The relative abundance of each annotated immune cell type is displayed for each sample (AR-1, AR-2, AR-3; Control-1, Control-2, Control-3). AR, allergic rhinitis; PBMC, peripheral blood mononuclear cell; t-SNE, t-distributed stochastic neighbor embedding.

Unsupervised clustering and cell-type annotation were performed on the single-cell RNA-seq dataset GSE180697. Ten major cell clusters were identified by t-SNE, including T cells, B cells, monocytes, and macrophages. After quality control, valid cell counts across all individual donors ranged from 200 to 990. CD4^+^ central memory T cells were detected in one control sample (GSM5468266), with a total of 53 cells accounting for 11.32% of all cells in this donor; this cell subset was undetectable in the other two control subjects and all three AR patients. Full per-donor quality control indicators and CD4^+^ central memory T-cell statistics are available in Supplementary Table S1. In this single-cell dataset, CD4^+^ central memory T cells could not be detected in any AR sample and were only recovered from a single control donor (Figure 6).

### 3.5. Construction of an Exploratory Six-Gene Molecular Signature Based on Immunomodulatory Genes

**Figure 7:**
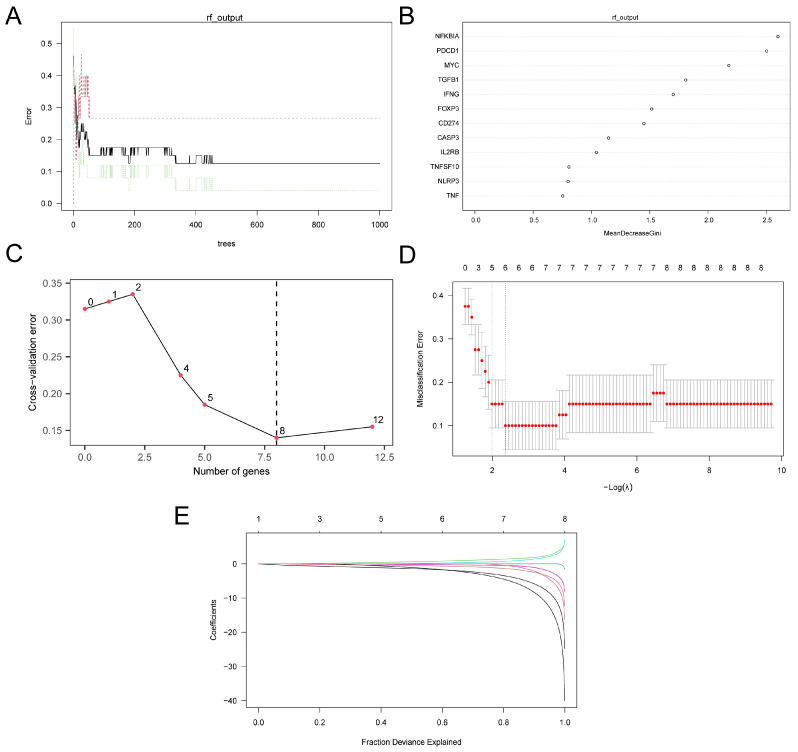
Screening of the diagnostic gene panel by machine learning. (**A**,**B**) RF gene importance ranking. The red horizontal dashed line in panel (**A**) represents the reference threshold for model error. (**C**) Cross-validation error curve based on 10-fold cross-validation for selecting optimal gene quantity. The numerical labels on the curve denote the number of genes included in the model; the dot at *n* = 8 stands for the optimal 8-gene panel screened by RF algorithm with the minimum cross-validation error. (**D**) LASSO regression curve with 10-fold cross-validation. The red horizontal dashed line marks the optimal λ value for model construction. (**E**) Coefficient profiles of the LASSO model. Each colored curve corresponds to the coefficient change of one individual gene with varying regularization parameters. RF, random forest; LASSO, least absolute shrinkage and selection operator.

A PPI network was constructed based on the 12 IMRDEGs. Random Forest and LASSO regression were applied to identify key diagnostic genes. Through this integrative feature selection process, six genes—*NFKBIA*, *PDCD1*, *MYC*, *IFNG*, *FOXP3*, and *CD274*—were identified as the core components of the diagnostic model (Figure 7).

**Figure 8:**
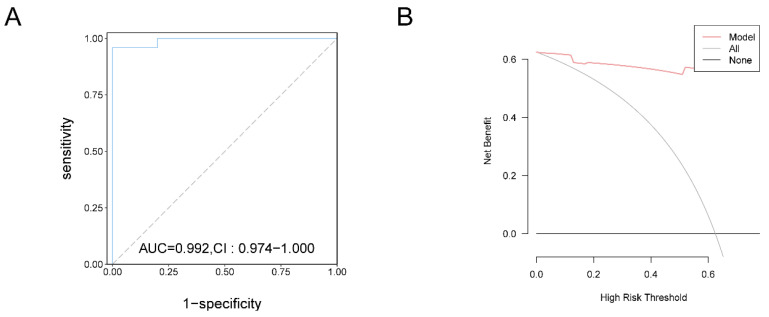
Apparent performance of the six-gene exploratory molecular signature in the training cohort (GSE75011). (**A**) ROC curve showing the discriminative ability of the six-gene model, with an AUC of 0.992 (CI: 0.974–1.000). The dashed lines represent the reference line of AUC = 0.5 (random diagnostic performance). (**B**) Exploratory DCA of the six-gene signature in the training cohort. ROC, receiver operating characteristic; AUC, area under the curve; CI, confidence intervals; DCA, decision curve analysis.

In the training cohort (GSE75011), the six-gene model achieved an AUC of 0.992 (95% CI: 0.974–1.000) and showed a favorable clinical net benefit in DCA (Figure 8).

**Figure 9:**
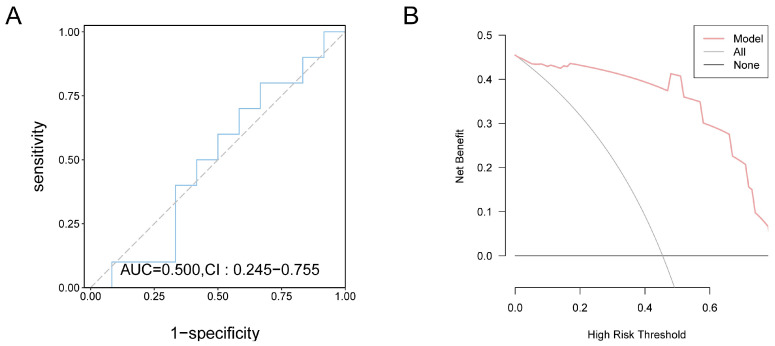
External validation of the six-gene exploratory molecular signature in the independent cohort (GSE44037). (**A**) ROC curve showing the discriminative ability in the validation dataset, with an AUC of 0.500 (CI: 0.245–0.755). The dashed lines represent the reference line of AUC = 0.5 (random diagnostic performance). (**B**) Exploratory DCA showing the net clinical benefit of the model across high-risk thresholds in the validation cohort. ROC, receiver operating characteristic; AUC, area under the curve; CI, confidence intervals; DCA, decision curve analysis.

In the external validation cohort (GSE44037), the model showed limited discriminative performance, with an AUC of 0.500 (95% CI: 0.245–0.755). Corresponding sensitivity and specificity metrics further indicated limited external generalization performance. DCA suggested a potential net benefit over a narrow range of risk thresholds, though the overall clinical utility was less robust than in the training set (Figure 9).

### 3.6. Functional Enrichment Analysis Indicates a Coordinated Immune Regulatory Alteration in Allergic Rhinitis

**Figure 10:**
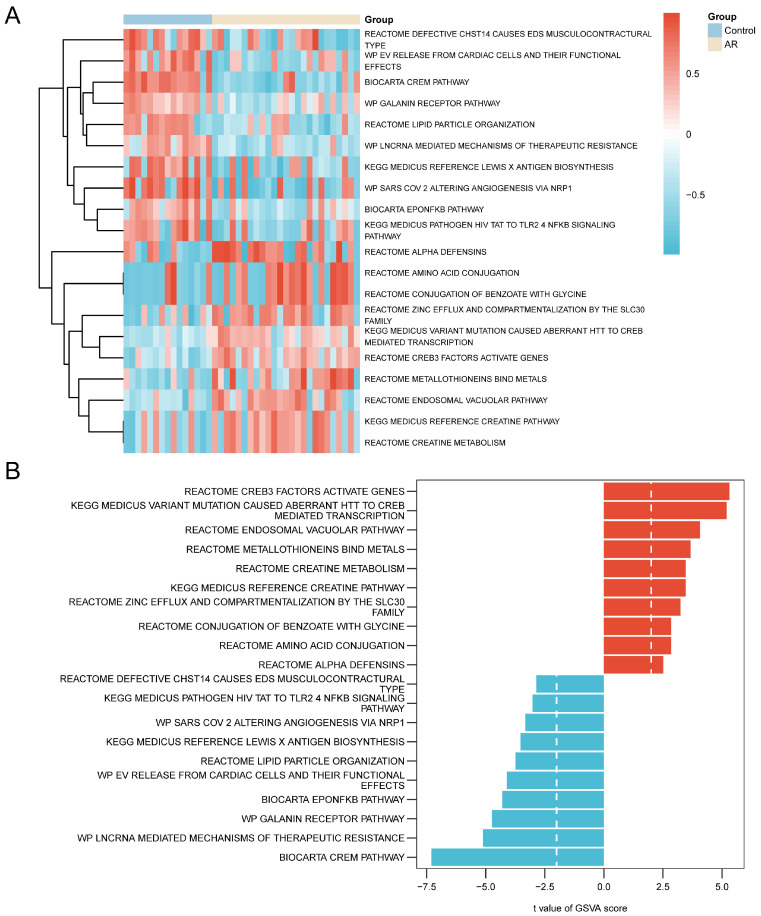
GSVA results between the AR and control groups in GSE75011. Heat map (**A**) and bidirectional bar graph (**B**) of GSVA results between AR and control of GSE75011. AR, allergic rhinitis, and GSVA, gene set variation analysis.

GSVA was performed to compare pathway enrichment between AR and control samples. AR samples exhibited significant enrichment of inflammatory response, *IL6-JAK-STAT3* signaling, complement activation, and coagulation cascades. In contrast, oxidative phosphorylation and peroxisome-related pathways were downregulated (Figure 10).

**Figure 11:**
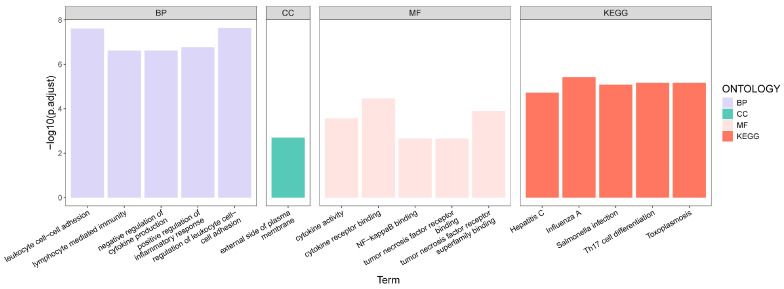
GO and KEGG enrichment analysis of hub immunomodulatory genes. GO (BP, CC, MF) and KEGG pathway enrichment analysis. The bar plot displays significantly enriched terms, with the y-axis representing the −log10-transformed adjusted *p*-values and the x-axis showing the corresponding functional/pathway terms. GO, Gene Ontology; BP, biological process; CC, cellular component; MF, molecular function; KEGG, Kyoto Encyclopedia of Genes and Genomes.

GO and KEGG analyses showed that hub genes were mainly enriched in leukocyte cell–cell adhesion, positive regulation of inflammatory response, lymphocyte-mediated immunity, Th17 cell differentiation, and Influenza A (Figure 11).

### 3.7. Cell–Cell Communication Analysis Suggests Abundance-Dependent Alterations in Immune Coordination in Allergic Rhinitis

Cell–cell communication analysis was performed using the CellChat framework to infer ligand–receptor-mediated interaction networks. The overall number and strength of intercellular interactions were substantially lower in AR than in controls.

**Figure 12:**
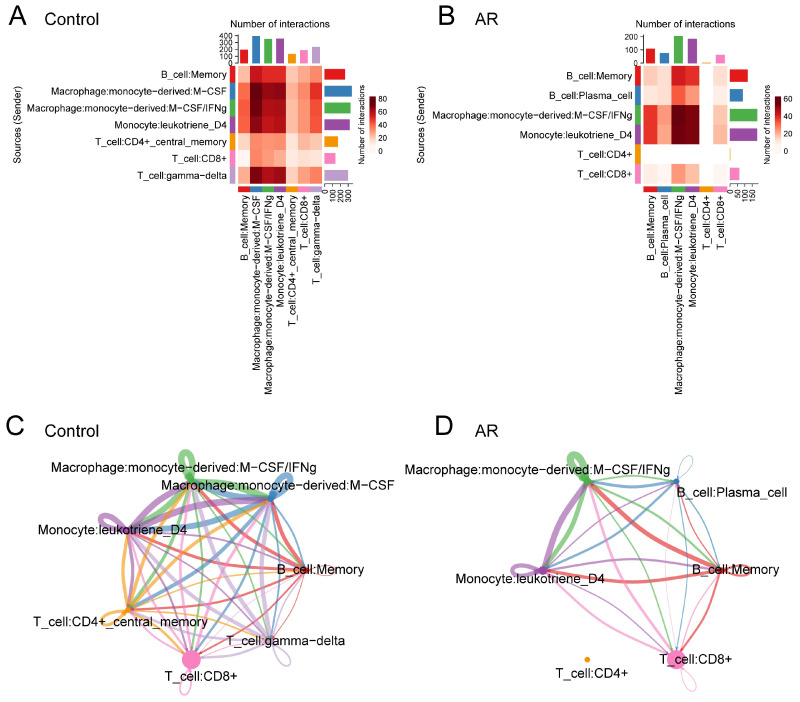
Global cellcell communication analysis. (**A**,**B**) Total interactions in control and AR. Reduced predicted outgoing signaling associated with CD4^+^ central memory T cells was observed in AR. (**C**,**D**) Communication patterns in control and AR groups. AR, allergic rhinitis.

Global comparison revealed a marked reduction in the number and strength of signaling from CD4^+^ central memory T cells in AR samples compared with controls (Figure 12).

Detailed analysis of cell-type-specific communication patterns showed that CD4^+^ central memory T cells contributed more predicted outgoing interactions in control samples. In AR samples, these predicted interactions were reduced in parallel with the non-detection of this cell subset, supporting a cautious interpretation of the altered communication landscape associated with changes in cell abundance.

**Figure 13:**
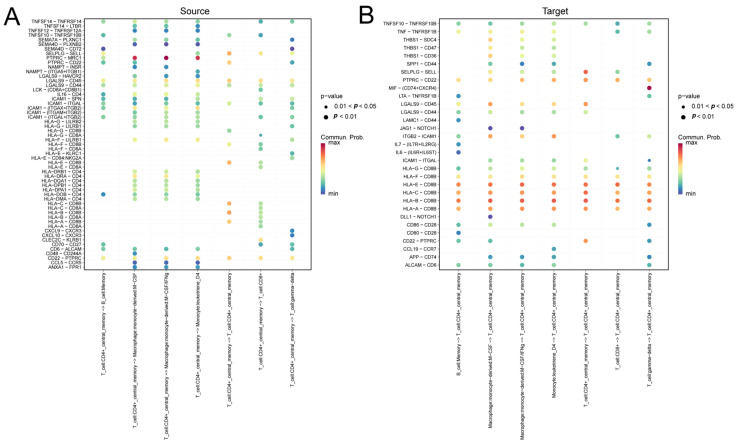
Significantly altered cell communication ligand–receptor pairs in GSE180697. (**A**) Bubble plot of ligand-receptor interaction strength in control samples. Bubble color represents interaction magnitude (red = strong, blue = weak), and bubble size indicates statistical significance of cell-cell communication. (**B**) Bubble plot of ligand-receptor interaction strength in allergic rhinitis samples, with consistent bubble color and size annotation rules as panel A. AR, Allergic rhinitis.

Furthermore, several signaling pathways involved in immune regulation and inflammatory control exhibited altered predicted activity, with relative differences in regulatory and persistence of pro-inflammatory interaction patterns (Figure 13).

### 3.8. Identification of in Silico Candidate from Drug-Repositioning Analysis in Allergic Rhinitis

Drug repositioning analysis was performed using the L1000CDS2 platform with DEGs from the GSE75011 dataset as input signatures to screen small-molecule compounds capable of reversing AR-related transcriptional changes.

All candidate compounds were first ranked by connectivity scores, and only those with an interaction ratio greater than 30% were retained according to their opposing regulatory effects on key genes.

A total of 50 perturbation signatures were obtained from the screening workflow. The connectivity scores of all retrieved records fluctuated between 0.0598 and 0.0872.

**Figure 14:**
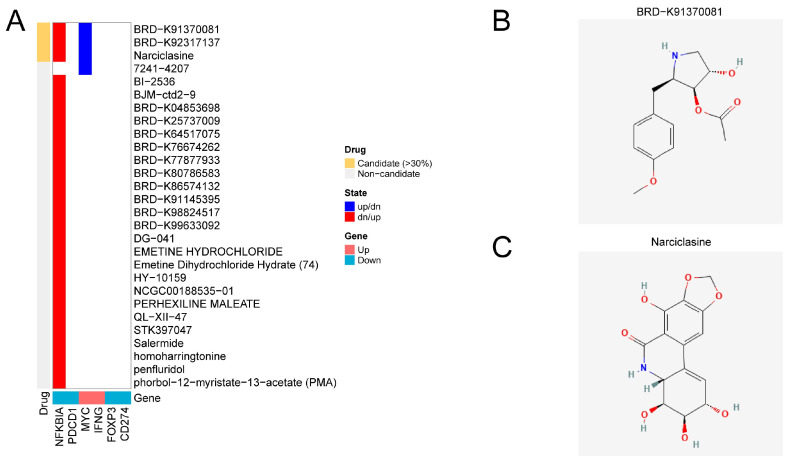
Candidate therapeutic compounds identified by drug repositioning. (**A**) Heatmap illustrating transcriptomic associations between L1000CDS2-screened candidate compounds and key disease genes. (**B**) Two-dimensional chemical structure of BRD-K91370081. (**C**) Two-dimensional chemical structure of Narciclasine. AR, allergic rhinitis.

Among the top candidates, narciclasine and BRD-K91370081 were identified as computationally predicted small-molecule candidates, which exhibited the possibility of reversing AR-related transcriptional signatures in L1000CDS2 analysis, which were predicted to regulate the expression of core genes, including *NFKBIA* and *MYC* (Figure 14). Narciclasine yielded a connectivity score of 0.0786 when tested on the HCC15 cell line with a treatment dose of 10.0 and incubation time of 6.0 h. BRD-K91370081 obtained a connectivity score of 0.0718 under the experimental context of HT29 cells at a dose of 10.0 and 6.0 h treatment.

## 4. Discussion

AR is characterized by pronounced immune heterogeneity, and increasing evidence suggests that disease persistence is associated with coordinated alterations across molecular, cellular, and regulatory immune networks. In the present study, we integrated bulk transcriptomic analysis, immune infiltration estimation, single-cell RNA sequencing, cell–cell communication analysis, machine learning, and drug repositioning to characterize the immunomodulatory landscape of AR. We identified 12 IMRDEGs, constructed an IM.Score, and observed coordinated alterations in immune pathways, immune cell composition, and intercellular communication. Notably, single-cell analysis further suggested altered recovery of CD4^+^ central memory T cells in this limited dataset. Collectively, these findings provide a multi-layered mechanistic framework for understanding immune dysregulation in AR and may inform future biomarker development and therapeutic exploration.

The identification of IMRDEGs provides molecular clues regarding immune dysregulation in AR. These genes represent major regulatory axes of immune function, including pro-inflammatory signaling (e.g., *TNF*, *IFNG*), immune tolerance and checkpoint pathways (e.g., *FOXP3*, *PDCD1/CD274*), and feedback control of inflammation (e.g., *NFKBIA*) [6,7,8,11]. The concurrent dysregulation of these genes indicates that AR is driven by the systemic collapse of immune regulatory networks rather than isolated excessive inflammation. This is consistent with emerging evidence that allergic diseases involve complex interactions among inflammatory, regulatory, and epithelial-immune pathways. Importantly, this gene set might support future molecular stratification of AR, given its involvement in multiple immune processes. Overall, the IMRDEG profile represents a candidate molecular layer associated with a broader immune network imbalance in AR.

Functional enrichment analyses provided pathway-level mechanistic validation for the coordinated immune dysregulation observed in AR. In our dataset, activation of inflammatory pathways was positively correlated with lower IM.Score in AR samples and divergent immune infiltration patterns between high- and low-IM.Score groups. The altered recovery of CD4^+^ central memory T cells was observed in parallel with these transcriptional and cellular patterns, but causal relationships cannot be inferred from the present analyses.

Enrichment of *IL6-JAK-STAT3* signaling and Th17-related pathways indicates the engagement of mixed inflammatory programs beyond canonical type 2 inflammation [4,5], while complement and coagulation cascade enrichment reflects widespread inflammatory amplification and tissue microenvironmental remodeling [12,13]. Additionally, suppressed oxidative phosphorylation suggests profound immunometabolic reprogramming that sustains chronic inflammatory activation [14]. These pathway-level alterations are biologically consistent with the observed depletion of CD4^+^ central memory T cells, since sustained inflammatory signaling and metabolic stress impair T-cell differentiation, survival, and functional stability, but further experimental studies are required to define their direct relationship with CD4^+^ central memory T-cell abundance [15]. Together, these results suggest that AR is associated with coordinated inflammatory, immunoregulatory, and metabolic alterations.

At the cellular level, single-cell analysis suggested altered recovery of CD4^+^ central memory T cells in AR. Central memory T cells play a critical role in maintaining long-term immune homeostasis by coordinating recall responses and balancing effector and regulatory pathways [16,17]. However, in the present dataset, CD4^+^ central memory T cells were recovered from only one control donor and were not detected in the remaining controls or AR samples, making formal donor-level statistical inference unstable. Therefore, this result should be interpreted as an exploratory cellular observation rather than definitive evidence of disease-specific loss. In the present analysis, altered CD4^+^ central memory T cells recovery occurred in parallel with lower IM.Score in AR samples, inflammatory pathway enrichment, immune infiltration differences, and attenuated intercellular communication involving this population. Communication strength values predicted by CellChat are heavily influenced by the size of each cell cluster and overall cellular composition within samples. Accordingly, weakened predicted signaling associated with CD4^+^ central memory T cells cannot be directly interpreted as impaired signal transduction capacity of individual cells. These findings suggest that CD4^+^ central memory T-cell abundance may represent a cellular correlate of the broader immunoregulatory disturbance observed in AR. Consistently, cell–cell communication analysis showed that CD4^+^ central memory T cells displayed more extensive outgoing interactions in control samples, whereas these communication signals were weakened in AR samples. This pattern indicates that AR-associated immune dysregulation may involve not only changes in immune cell composition but also alterations in coordinated signaling among immune cell populations [18]. Further experimental studies are warranted to determine whether the reduction in CD4^+^ central memory T cells is a driver, consequence, or compensatory feature of chronic allergic inflammation. Notably, these transcriptome-based cellular observations still require validation using independent clinical specimens and functional laboratory experiments.

The IM.Score developed in this study provides a quantitative measure of immune regulatory status in AR by integrating coordinated expression changes in immune-related genes. Unlike single-gene biomarkers, IM.Score may better capture the balance between inflammatory activation and regulatory control, thereby reflecting immune heterogeneity [19]. Building on the IM.Score-based immunomodulatory signature, we further selected key genes to construct a six-gene exploratory molecular signature via machine learning. The six-gene model, consisting of *NFKBIA*, *PDCD1*, *MYC*, *IFNG*, *FOXP3*, and *CD274*, showed favorable discriminative performance in the training cohort, yet the AUC value reached only 0.500 in external validation, indicating that its performance failed to support stable cross-cohort generalizability and reflecting limited diagnostic capacity in independent patient populations. The AUC of 0.500 observed in the external cohort suggests that this panel lacks robust diagnostic discriminatory capacity when applied to an unrelated patient population. Accordingly, the six-gene signature identified herein should be interpreted merely as an exploratory transcriptomic feature rather than a tool ready for direct clinical diagnostic use.

The most prominent contributor to the weakened predictive efficacy between training and validation cohorts is the inherent tissue heterogeneity across datasets. The training cohort GSE75011 consists of peripheral blood samples. It mainly captures systemic transcriptional alterations of immune responses linked to AR. The validation cohort GSE44037 contains nasal epithelial cells. These specimens better recapitulate local inflammatory reactions within the upper airway mucosa and epithelial compartments. Cellular composition, baseline transcriptional profiles, and disease-associated expression patterns vary substantially across distinct tissue types. Gene signatures trained using peripheral blood may not sustain consistent discriminatory performance when applied to nasal epithelial samples. Beyond cross-tissue divergence, other confounding factors further impair external generalization, including model overfitting to the limited training dataset, inconsistent detection platforms, insufficient sample sizes across both cohorts, residual uncorrected technical batch effects, and broad inter-individual biological heterogeneity among AR patients. Based on the current findings, this gene panel serves primarily as a candidate clue for follow-up mechanistic investigations and validation in separate cohorts, and its clinical translational value requires further rigorous evaluation using larger, well-designed independent cohorts with consistent tissue sources. Despite these limitations on cross-cohort predictive power, the six selected genes remain biologically interpretable, as they participate in core regulatory cascades covering inflammatory signaling, immune checkpoint modulation, and T-cell immune homeostasis.

From a translational perspective, IM.Score and the gene-based molecular signature might serve as tools for patient stratification, disease monitoring, and therapeutic guidance. Future studies should validate their clinical application potential in larger, well-characterized cohorts and assess whether dynamic changes in IM.Score are associated with disease severity, symptom burden, or treatment response.

Recently, the application of artificial intelligence-assisted decision-making in rhinologic disorders and precision medicine has attracted widespread attention. Such computational tools have potential for integrating complex clinical and multi-omics molecular datasets, yet substantial limitations remain regarding evidence quality, model interpretability, clinical real-world validation, and safety supervision. Therefore, the multi-omics molecular signatures uncovered in this work could be combined with artificial intelligence-assisted computational pipelines in future translational research, while all clinical translation attempts must rely on rigorous multi-center experimental and clinical verification [20].

Drug repositioning analysis identified computationally predicted candidate small-molecule compounds capable of modulating key regulatory genes, including *NFKBIA* and *MYC*. Narciclasine has been shown to exert anti-inflammatory effects by suppressing NF-κB signaling and reducing cytokine production [21], while *MYC* is recognized as a regulator of immune cell activation and metabolic reprogramming [22]. These results suggest that these small molecules can serve as candidate clues for subsequent drug screening and mechanistic verification. Compared with conventional therapies that primarily suppress downstream inflammation, such approaches might help restore immune regulatory balance. However, these predictions require experimental validation, and future studies should explore their efficacy in relevant models. Notably, the involvement of multiple interconnected pathways suggests that combination strategies targeting immune regulatory networks might offer improved therapeutic outcomes.

This study has several limitations. First, the findings are primarily based on bioinformatics analyses using publicly available datasets and, therefore, lack experimental validation in clinical samples or in vivo models. Second, the training cohort (peripheral blood) and external validation cohort (nasal epithelial cells) were obtained from different tissue compartments, which inherently leads to substantial tissue-specific differences in baseline gene expression profiles. This tissue heterogeneity likely constitutes a major driver of the poor discriminative performance observed in the external validation set. Even though we standardized all bulk expression matrices and verified normalization effectiveness using boxplots and PCA analyses, residual batch effects stemming from inconsistent research cohorts, detection platforms, and tissue types could still compromise the model’s predictive capacity. Third, single-cell RNA-seq analysis only included three AR patients and three healthy controls. The limited sample size cannot adequately represent the broad inter-individual immune heterogeneity of AR patients. This finding shall only be treated as a preliminary observation and cannot independently support the conclusion that CD4^+^ central memory T-cell depletion acts as a stable cellular signature or core pathogenic mechanism of AR. Further validation based on larger single-cell cohorts is required to corroborate this potential trend. Fourth, the drug repositioning analysis was conducted solely based on reverse transcriptomic signature matching. Accordingly, relevant findings should be regarded as preliminary predictive outcomes. Future studies should integrate prospective clinical cohorts, matched nasal and peripheral immune samples, mechanistic experiments, and longitudinal follow-up to firmly establish the biological relevance and clinical applicability of the IM.Score, six-gene signature, CD4^+^ central memory T-cell alterations, and potential candidate biomarkers and therapeutic clues requiring further experimental validation.

In conclusion, this study presents an integrative multi-omics framework that delineates immune dysregulation in AR at molecular, pathway, cellular, and network levels. Our findings establish a hypothesis-generating model in which AR is associated with coordinated disturbance of the immune regulatory network, with altered CD4^+^ central memory T-cell abundance serving as a potential cellular correlate linking molecular dysregulation to system-level immune imbalance. These insights not only deepen our understanding of AR pathogenesis but also deliver potential candidate biomarkers and therapeutic clues requiring further experimental validation for future research on precision management of AR. Future work will focus on validating these findings in prospective cohorts and exploring the translational potential of the identified signatures and compounds.

## 5. Conclusions

This integrative bioinformatics study systematically delineated the immunomodulatory landscape of allergic rhinitis by combining bulk transcriptomics, single-cell RNA sequencing, machine learning, and drug repositioning approaches. We identified 12 IMRDEGs and constructed an IM.Score based on these genes and observed distinct IM.Score values between the AR and control samples. Immune infiltration analysis revealed IM.Score-associated immune microenvironmental differences. At a single-cell resolution, we did not detect CD4^+^ central memory T cells in the three AR samples and observed the recovery of this subset in only one control donor, a tentative cellular observation that should be interpreted cautiously because of the small cohort size and sparse cell recovery.

Furthermore, we developed a six-gene exploratory molecular signature that achieved excellent discriminative performance in the training cohort (GSE75011), while external validation in an independent dataset (GSE44037) showed limited predictive performance, likely due to tissue source heterogeneity, platform differences, sample-size limitations, and potential overfitting. We identified narciclasine and BRD-K91370081 as computationally predicted small-molecule candidates with potential to reverse pathogenic transcriptional profiles in AR. These candidate therapeutic clues remain to be further verified via molecular docking, target analysis, and laboratory experimental research. Collectively, our findings support an exploratory model in which allergic rhinitis is associated with coordinated immune regulatory disturbance, with altered CD4^+^ central memory T-cell abundance representing a potential immune cellular correlate of molecular and network-level immune imbalance. This study proposes candidate molecular, cellular, and pharmacological clues that require further confirmation through laboratory experiments and clinical cohort investigations.

## Data Availability

The datasets analyzed in this study are publicly available in the Gene Expression Omnibus (GEO) database (https://www.ncbi.nlm.nih.gov/geo/, accessed on 23 March 2026) under accession numbers GSE75011, GSE44037, and GSE180697. All data used in this study are accessible without restriction.

## Supplementary Materials

The following supporting information can be downloaded at https://www.mdpi.com/article/10.3390/biomedicines14071541/s1. Table S1. Per-donor quality control metrics and CD4^+^ central memory T-cell proportions in the single-cell dataset.

## Abbreviations

The following abbreviations are used in this manuscript:

AUC	Area Under the Curve
AR	Allergic Rhinitis
BP	Biological Process
CC	Cellular Component
CI	Confidence Intervals
DCA	Decision Curve Analysis
DEGs	Differentially Expressed Genes
FDR	False Discovery Rate
GEO	Gene Expression Omnibus
GO	Gene Ontology
GSEA	Gene Set Enrichment Analysis
ssGSEA	Single-Sample Gene Set Enrichment Analysis
GSVA	Gene Set Variation Analysis
IM.Score	Immunomodulatory Score
IMRDEGs	Immunomodulatory-Related Differentially Expressed Genes
IMRGs	Immunomodulatory-Related Genes
KEGG	Kyoto Encyclopedia of Genes and Genomes
LASSO	Least Absolute Shrinkage and Selection Operator
MF	Molecular Function
PBMC	Peripheral Blood Mononuclear Cell
PCA	Principal Component Analysis
PPI	Protein–Protein Interaction
QC	Quality Control
RF	Random Forest
RMSE	Root Mean Square Error
ROC	Receiver Operating Characteristic
t-SNE	t-Distributed Stochastic Neighbor Embedding
