# Supplementary material for: Integrative Multi-Omics Analysis Characterizes Immune Dysregulation and Altered CD4+ Central Memory T-Cell Abundance in Allergic Rhinitis
Source: Biomedicines. 2026 Jul 9;14(7):1541. doi: 10.3390/biomedicines14071541 (PMC13405808; doi:10.3390/biomedicines14071541)
Supplement: Supplementary file 1 [file biomedicines-14-01541-s001.zip › biomedicines-4370774-supplementary.pdf]

Supplementary Table S1. Per-donor quality control metrics and CD4$^+$ central memory T-cell proportions in the single-cell dataset

Sample_ID	Group	Post_QC_Cells	Median_Genes	Median_UMI	Median_MT	MT_Threshold	Clustering_Resolution	CD4_CM_Count	CD4_CM_Percentage
GSM5468249	Control	592	3828	14288.5	3.14	<25%	0.6	0	0
GSM5468266	Control	468	4087.5	14527.5	2.61	<25%	0.6	53	11.32
GSM5468272	Control	990	3681	12940.5	2.48	<25%	0.6	0	0
GSM5468279	AR	406	3374	9984.5	6.14	<25%	0.6	0	0
GSM5468292	AR	356	3074	9398	7.00	<25%	0.6	0	0
GSM5468305	AR	200	3104	9159.5	7.26	<25%	0.6	0	0

Detailed quality control metrics and CD4$^+$ central memory T cell statistics for each individual donor in the single-cell RNA-seq dataset GSE180697. The mitochondrial read proportion threshold was set to <25%, and clustering resolution was fixed at 0.6 for all samples. CD4$^+$ central memory T cells were detected only in one control sample (GSM5468266), with no detection in the remaining 5 donors.

Abbreviations: AR, allergic rhinitis; MT, mitochondrial gene; UMI, unique molecular identifier; QC, quality control; CD4_CM, CD4$^+$ central memory T cell
